# Reduction of facial pigmentation of melasma by topical lignin peroxidase: A novel fast-acting skin-lightening agent

**DOI:** 10.3892/etm.2014.2118

**Published:** 2014-12-08

**Authors:** SHAO-MIN ZHONG, NAN SUN, HUI-XIAN LIU, YUE-QING NIU, YAN WU

**Affiliations:** Department of Dermatology, Peking University First Hospital, Beijing 100034, P.R. China

**Keywords:** lignin peroxidase, melanin, melasma, skin lightening, emulsions, statistics

## Abstract

The aim of the present study was to evaluate the efficacy and safety of lignin peroxidase (LIP) as a skin-lightening agent in patients with melasma. A self-controlled clinical study was performed in 31 women who had melasma on both sides of the face. This study involved 8 weeks of a full-face product treatment. The skin color was measured at days 0, 7, 28 and 56 using a chromameter on the forehead and cheeks. Standardized digital photographic images of each side of the face of all subjects were captured by a complexion analysis system. Clinical scores of the pigmentation were determined by two dermatologists. After using the LIP whitening lotion for 7 days, the luminance (L*) values of the melasma and the normal skin were significantly increased from baseline. The L* values continued to increase at days 28 and 56. The melasma area severity index (MASI) score was statistically decreased after 28 days of treatment. No treatment-related adverse events were observed. LIP whitening lotion was able to eliminate the skin pigmentation after 7 days of treatment, and provides a completely innovative approach to rapid skin lightening. The LIP whitening lotion exhibited good compatibility and was well tolerated.

## Introduction

Melasma is a common, acquired hyperpigmentary disorder predominantly affecting dark-skinned populations, which has a severe impact on the quality of life of a patient (1). Its long-term management remains a significant therapeutic challenge for dermatologists (2). Melanin is a durable compound that is hard to destroy (3). Therefore, most available treatments target the formation of melanin by blocking its biosynthesis, which is a slow and inefficient process. Skin lightening actives with new mechanisms are now becoming of interest (1,4). Lignin peroxidase (LIP) is a purified active enzyme derived from the fermented fungus *Phanerochaete chrysosporium* under controlled sterility conditions. The molecular structure of lignin is similar to that of melanin, and a previous study confirmed that LIP has the potential to improve skin tone by reducing eumelanin. This enzyme is enabled by the pulse feeding of H_2_O_2_ and breaks down melanin, creating a fast-acting melanin-eliminating effect (5). The present study aimed to investigate the efficacy and tolerability of a novel whitening lotion containing LIP in the treatment of melasma.

## Materials and methods

### Study design

This was an 8-week, single-center, open label, self-controlled prospective study. The subjects that participated in the study were healthy Chinese women, 25–55 years of age (mean ± SD, 42.12 ± 8.37 years), who had melasma involving the face. Only patients who had not undergone topical treatment with hydroquinone cream, corticosteroids, or any other de-pigmenting medication or any other photosensitizing medication within 3 months prior to enrollment or who had not undergone light-to-medium peels or microdermabrasion within 6 months prior to study enrollment were included. Pregnant women or women taking birth control medication, hormone replacement therapy or any other hormone-altering medication 6 months prior to study recruitment were further excluded. This study was conducted at Beijing, China, in March to May 2012 in accordance with the WHO guidelines for good clinical practice (GCP) for trials on pharmaceutical products (6). The protocol was reviewed and approved by Ethics Committee of Peking University First Hospital (Beijing, China). Each subject provided signed informed consent.

### Treatment

Following a washout period of 2 weeks with a gentle cleanser and a sun protection factor 30+ (SPF30+) sunscreen, all subjects were given a study products set, which included a cleanser, whitening lotion, activator lotion and sunscreen. All test products were provided by Syneron Medical Inc. (Irvine, CA, USA). The subjects were instructed to clean their face using the cleanser twice a day. After cleaning, the subjects were instructed to cover the whole face with the whitening lotion and wait 1 min before applying the activator lotion, which allowed the enzyme to attain a balance at the appropriate pH value. Subjects applied the study products twice daily for 8 weeks. Subjects were permitted to continue their usual facial treatment regimen that did not contain active ingredients (e.g., α-hydroxy acid, salicylic acid, vitamin A or arbutin). In addition, the patients were instructed to use SPF30+ sunscreen during the day for the duration of the study course.

### Assessment regimen and instrumental measurements

The subjects were photographed and measurements taken four times during the study course: prior to the treatment (day 0), and one week (day 7), 4 weeks (day 28) and 8 weeks (day 56; end of treatment period) after the first treatment.

### Test environment

Prior to each measurement, subjects washed their face with the assigned facial cleanser and underwent equilibration for 30 min in a room with controlled temperature (21–24°C) and relative humidity (30–50%).

### Spectrophotometric analysis

The forehead and cheeks were assessed for pigment lightening using the CM-2500d Spectrophotometer (Minolta, Tokyo, Japan) as a chromameter as described previously (7). The L* value (luminance) defines the relative lightness ranging from total black (L*=0) to total white (L*=100); the a* value represents the balance between red (positive value) and green (negative value). Three consecutive measurements were taken at each site and the average of three measured values was calculated and considered to be the absolute value.

### Facial imaging

As shown in Fig. 2, standardized images of each side of the face of all subjects were captured under the same visible light and UV light conditions with the Visia^®^ (Canfield Imaging Systems, Fairfield, NJ, USA) complexion analysis system at different time points, using a previously described method (8).

### Evaluation of changes in pigmentation

Evaluations of melasma area severity index (MASI) scores were performed at baseline and during the test period by two dermatologists independently according to the method described previously (9). The average of the two assessments was used for analysis.

### Assessment of adverse effects

During the test period, skin irritation findings and adverse effects including erythema, desquamation, burning/stinging sensation and dryness were evaluated by dermatologists using a 0–3 Likert scale: 0, none; 1, mild; 2, moderate; and 3, severe.

### Statistical analysis

Statistical analysis was conducted using SPSS software, version 12.0 (SPSS, Inc., Chicago, IL, USA). Data are presented as the mean ± SD of triplicate measurements. Data were analyzed and compared prior to and following the treatment using analysis of variance (ANOVA) or Dunnett’s comparison for parametric data and/or the Friedman or Wilcoxon test for non-parametric data. For all analyses, P<0.05 was considered to indicate a statistically significant difference.

## Results

### Study completion

The study included 33 melasma patients in total and 31 patients completed the study. Two patients were lost at day 7 due to the adverse effects not being following up, and so were excluded from the data analysis.

### Skin lightness measurements

**Figure 1:**
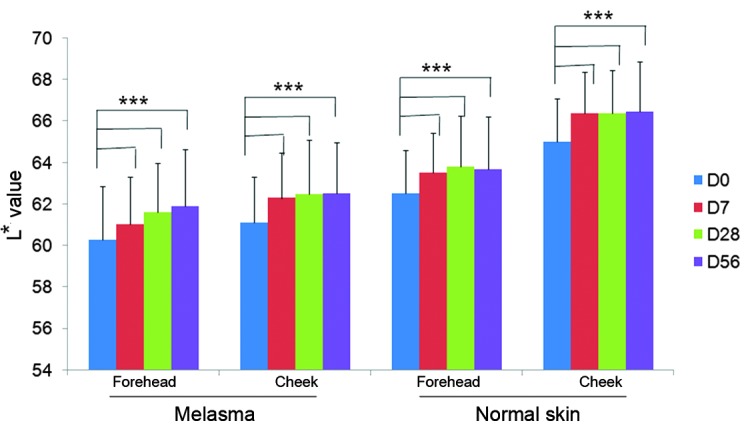
Skin lightness measurements at different time points of treatment. ^***^P<0.001. D, day; L, luminance.

**Figure 2:**
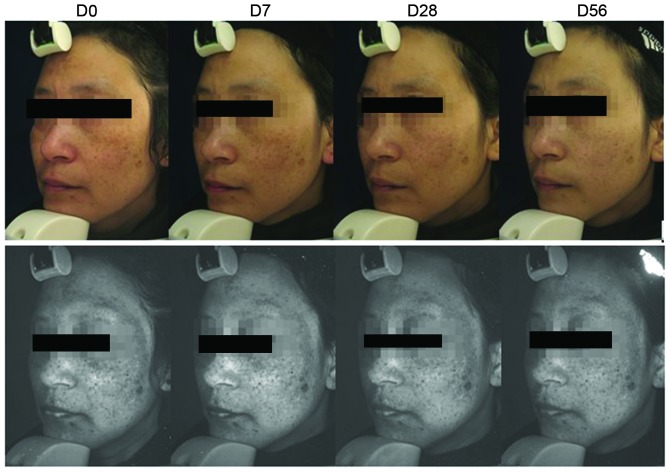
Normal and UV pictures of a patient prior to and following treatment at different time points. D, day.

At baseline, the mean L* value representing the skin lightness of facial subareas with melasma was 60.57±2.39 while the respective value of facial subareas without melasma was 64.16±2.73 (P<0.05; ANOVA). Following 7 days of product application, the mean values increased significantly with mean L* values of 61.42±2.46 and 65.41±2.62 for melasma and non-melasma subareas, respectively (P<0.05; ANOVA). The mean values representing skin lightness at different subareas of the face continued to increase progressively at day 28 and further on day 56. The difference in L* values was statistically significant between all time points (Fig. 1).

### Skin redness measurements

**Figure 3:**
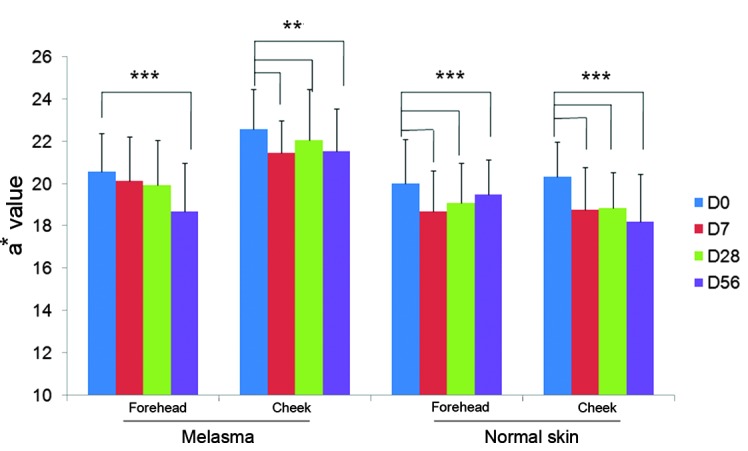
Skin redness measurements at different time points of treatment. ^***^P<0.001. D, day.

At baseline, the mean values representing the redness of the skin (a*) were higher at all facial subareas affected by melasma as compared with those without melasma. Following 7 days of study product application, all mean values representing skin redness decreased significantly compared with their respective baseline values (P<0.05; ANOVA). The mean values of subareas with and without melasma were also significantly reduced compared with baseline values at days 28 and 56 (Fig. 3).

### Clinical evaluation of melasma severity

**Figure 4:**
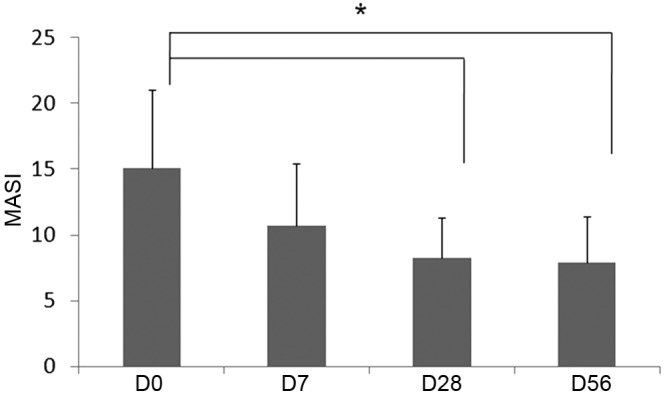
Changes in melasma area severity index (MASI) scores. ^*^P<0.05. D, day.

Physician assessment of melasma severity demonstrated improvement following 1, 4 and 8 weeks of product application. The difference in severity from the baseline value was statistically significant at 4 and 8 weeks after the initiation of treatment (Fig. 4).

### Safety evaluation

The treatment was tolerated well by all subjects. No adverse events and/or complications occurred during the period of product application.

## Discussion

Melasma is a common skin pigment disorder that sometimes has a severe emotional effect on the patient. The etiology of melasma remains unclear and current treatments have varying effectiveness on depigmentation (10). The gold standard of medical therapy is 4% hydroquinone, as a monotherapy or in combination with other depigmenting agents such as corticosteroids and/or retinoids (2). Over-the-counter (OTC) hydroquinone has been banned in Europe and Asian countries, since oxidized hydroquinone is toxic to melanocytes. In the USA, the Food and Drug Administration is currently evaluating the status of OTC hydroquinone and prescription products that are sold without approval (11).

This regulatory arena has created a requirement for pigment lightening alternatives with high tolerability and efficacy for worldwide use.

Melanin is composed of covalently linked indoles; it is a heterogeneous polymer formed from dihydroxyindole units (12). Its structure is similar to that of lignin or coal, in which polymers are composed of indolic or phenolic subunits. White-rot fungus, *Phanerochaete chrysosporium*, causes decolorization and depolymerization of low-grade coal under culture conditions that facilitate the mineralization of lignin. These actions of lignin-degrading enzymes make it possible to use them to decolorize melanin, which possesses a structure similar to those of coal or lignin (13). A previous study confirmed that the enzyme LIP has the potential to improve skin tone by reducing eumelanin (5). LIP is produced extracellularly during submerged fermentation of the fungus *Phanerochaete chrysosporium* and may be purified from the fermented liquid medium (5). Following final formulation and packaging, the product contains an active enzyme component and an activator component H_2_O_2_. The combination of the two formulations creates a temporary reaction that results in a targeted and time-limited catalysis of eumelanin degradation in the epidermis.

The present clinical study demonstrated that the active enzyme LIP, when formulated and used together with activator H_2_O_2_ is effective in improving skin pigmentation. Eight weeks of the twice daily regimen increased skin lightness and markedly decreased dyspigmentation in the facial subareas affected by melasma and those without melasma. Most importantly, effective results were observed as early as 7 days after the start of product application, suggesting a rapid response to treatment. Skin brightness at all areas increased gradually over the treatment period and became most significant 56 days after the initiation of treatment. It should be emphasized that the product was tolerated well by all subjects and no associated adverse events occurred during the treatment period, which should allow its continued use over a prolonged period of time.

It is concluded that LIP represents a novel skin-lightening product that provides a completely innovative advanced approach to achieve a rapid-acting skin-whitening effect. The results suggest that the skin-brightening complex, when appropriately formulated for topical use and used together with daily sun protection, is a valuable alternative to existing whitening products. However, the present study was a preliminary clinical study with limited subject numbers. The superiority of this depigmenting effect would be better assessed in a parallel group, double-blind, vehicle and active comparator-controlled clinical study with a higher number of volunteers (14).
